# TIGIT in cancer: from mechanism of action to promising immunotherapeutic strategies

**DOI:** 10.1038/s41419-025-07984-4

**Published:** 2025-09-01

**Authors:** Haozhe Cui, Mawieh Hamad, Eyad Elkord

**Affiliations:** 1 Department of Biosciences and Bioinformatics & Suzhou Municipal Key Lab of Biomedical Sciences and Translational Immunology, School of Science, Xi’an Jiaotong-Liverpool University, Suzhou, Jiangsu China; 2 College of Health Sciences, University of Sharjah, Sharjah, United Arab Emirates; 3 Biomedical Research Center, School of Science, Engineering and Environment, University of Salford, Manchester, UK

**Keywords:** Cancer immunotherapy, Tumour immunology

## Abstract

TIGIT immune checkpoint (IC) has attracted great interest in recent years. It belongs to the PVR-like protein family, and it inhibits T and NK cell cytotoxic activities. TIGIT mediates its inhibitory effect by direct signaling through the cytoplasmic tail, CD155-mediated inhibition, or competition with the immune-activating receptor CD226. Preclinical observations from studies involving TIGIT-specific blocking monoclonal antibodies (mAbs) are promising, but the results of the clinical trials using anti-TIGIT mAb monotherapy were not favorable, which prompted a focus on combinational therapies. Some alternative approaches have the potential to avoid limitations, including low penetration, immunogenicity and safety of mAbs. This review addresses the mechanisms underlying TIGIT-mediated immune suppression. Additionally, promising immunotherapeutic approaches against TIGIT, including co-inhibition of TIGIT with other ICs, using small molecule inhibitors, blocking the TIGIT/PVR pathway using CAR-T cells and the current state of clinical trials as well as future directions, are discussed.

## Facts

- TIGIT can suppress multiple mechanisms of anti-tumor immunity.
- TIGIT synergizes with PD-1 at the molecular level to inhibit CD226.
- Anti-TIGIT/PD-1 bsAbs demonstrate therapeutic advantages over mAb combinations.
- Small molecule compounds and peptides are potential alternatives to anti-TIGIT mAbs.
- Designed CAR targeting TIGIT can enhance tumor killing in CAR-T therapy.

## Open questions

- How to better define the co-use of other immunotherapies with TIGIT blockade to overcome the suppressive immune microenvironment?
- What are the biomarkers for anti-TIGIT therapy?
- Since immunomodulatory receptors such as TIGIT have multiple ligands, how can a larger immunomodulatory network be considered in therapy?

## Introduction

The immune system in humans and other mammals is tasked with clearing foreign pathogens and autologous abnormal (tumor) cells [1–3]. Continuous clearance of tumor cells is often hampered by the fact that some cells develop immune escape mechanisms under the selective pressure of the immune system [4]. The expression of immune checkpoints (ICs) is a prime example of this phenomenon. ICs belong to a class of receptors that, under normal conditions, hinder the adaptive immune response to prevent autoimmunity [5]. IC receptors are expressed on a variety of immune cells, such as T lymphocytes and natural killer (NK) cells [6]. As their ligands are also expressed on tumor cells, their engagement tends to inhibit a wide range of immune cells [5, 7, 8].

Several monoclonal antibodies (mAbs) that inhibit immune checkpoints (immune checkpoint inhibitors, ICIs) have been developed and approved for clinical use in several cancers. ICIs have proven beneficial in mouse models and human clinical trials [9–15]. Early promising results have served as an impetus for extensive work on identifying additional ICs and developing ICIs to target them in different cancers. Although treatment with ICIs has yielded some promising results in preclinical and clinical testing, a significant percentage (>50%) of the patients still respond poorly to ICIs or do not respond at all [16–18]. Deficiency of neoantigens due to low tumor mutational burden (TMB) and downregulation of MHC-I molecules can lead to insufficient tumor antigenicity, resulting in defective antigen presentation [18, 19]. Compensatory mechanisms between ICs, inadequate T-cell infiltration (cold tumors) and accumulation of Tregs at tumor sites can also cause low response rates to ICIs [20]. Despite these setbacks, interest in finding alternative novel ICs and ICIs continues to grow.

T cell immunoglobulin and ITIM domain (TIGIT), also known as WUCAM, Vstm3, or VSIG9, is a novel IC with potential to enhance clinical outcomes in patients [21, 22]. TIGIT was identified in 2009 as a member of the poliovirus receptor-related (PVR)-like protein family and has been shown to play a central role in tumor immunity and is a promising target in immunotherapy [21, 23]. Clinical trials of some mAbs that target TIGIT did not reproduce the promising preclinical data; in fact, administration of the anti-TIGIT mAbs alone often was associated with severe treatment-related adverse events (TAREs) and low objective response rate (ORR) [24, 25]. As a result, there has been a shift in focus from anti-TIGIT mAbs monotherapy to ICs co-inhibition and other modalities, which could harness the potential therapeutic value of targeting TIGIT. This review will first discuss the mechanisms underlying the inhibitory effect of TIGIT on tumor-related immunity and combination immunotherapeutic strategies, then discuss current approaches targeting TIGIT beyond mAbs monotherapy.

## Biology of TIGIT

### Expression and function

TIGIT is expressed by various types of T cells, including activated CD4^+^ T helper cells, CD8^+^ cytotoxic T cells, γδ T cells and T regulatory cells (Tregs), but not on naive T cells [22, 26, 27]. TIGIT is also not expressed on CD11c^+^ dendritic cells, CD68^+^ macrophages, or CD20^+^ B lymphocytes [28–30]. A study compared TIGIT and PD-1 expression patterns across healthy lymphoid tissues, inflamed tissues, and tumor tissues. Results revealed co-expression in over 70% of TIGIT^+^ cells with PD-1, while reciprocally, >90% of PD-1^+^ cells co-expressed TIGIT, suggesting a potential for dual inhibitory targeting [28]. It must be noted that the same cell type may express different levels of TIGIT depending on cellular localization. Greater than 95% of CD4^+^ T cells in lymphoid follicles expressed TIGIT relative to only <50% of CD4^+^ T cells residing in interfollicular compartments [28]. Interestingly, TIGIT expression levels in peripheral blood and tumor tissues in patients with primary breast cancer (PBC) seem to be related to the age of the patient, but not the tumor size or lymph node metastasis [31]. Tumor-infiltrating lymphocytes (TILs) were reported to express significantly higher levels of TIGIT than peripheral T cells [32, 33]. Some investigators have suggested that TIGIT overexpression could be closely associated with T-cell exhaustion, occurring in advanced tumor stages and following cancer cell antigen exposure [34, 35]. TIGIT expression levels also correlate with poor prognosis in many tumors, suggesting an important role in tumor progression, invasiveness, and/or metastasis [36, 37]. TIGIT knockout studies have reported a reversal of T and NK cell exhaustion coupled with enhanced anti-tumor immunity [38–40].

Besides its ability to inhibit tumor killing by effector T and NK cells, TIGIT is involved in regulating the function of Tregs. In vitro positive stimulation of Tregs expressing TIGIT can upregulate interleukin 10 (IL-10) and fibrinogen-like protein 2 (FGL2), which are both immunosuppressive factors inhibiting T cells [41]. Previous work has also shown that TIGIT mediates inhibitory signals by suppression of IFN-γ production and downregulation T-bet, a transcription factor (TF) influencing Treg function in type 1 inflammatory responses, which is regulated by IFN-γ. TIGIT also promotes the nuclear localization of FOXO1, which is a broadly expressed TF and important for NK/CD8^+^ T-cell activity and differentiation of Tregs [42]. This suggests that TIGIT-delivered signals promote the suppressive function of Tregs on effector T cells. Unlike PD-1/CTLA-4, inhibition of TIGIT in tumor therapy restores the immune function of T cells through multiple pathways [43].

### TIGIT structure and its ligands

**Fig. 1:**
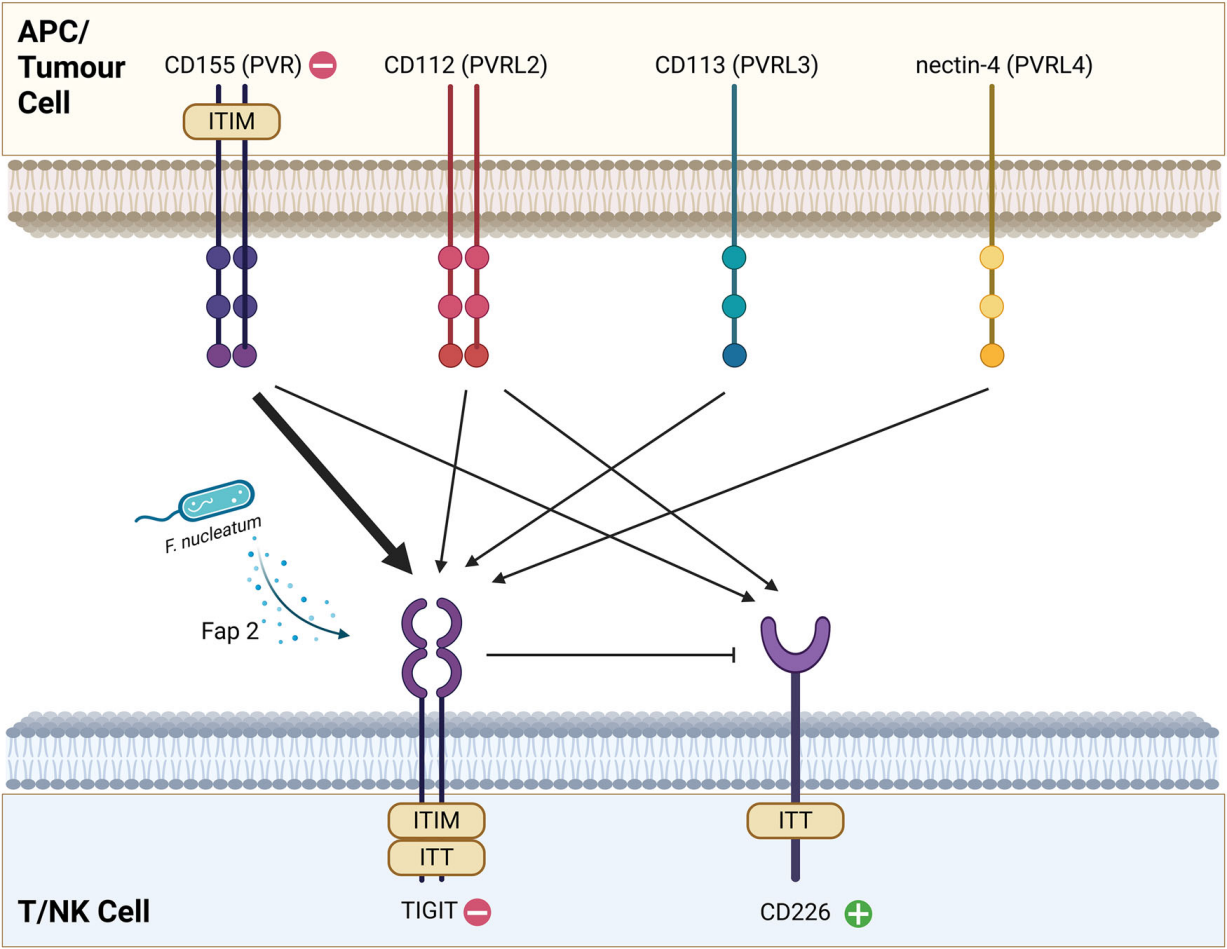
Interaction of TIGIT and CD226 with their ligands. TIGIT and CD226 are expressed in T and NK cells and appear as transmembrane proteins. Their ligands are expressed in antigen-presenting cells or tumor cells and are also localized to the cell membrane. TIGIT has an immunoreceptor tyrosine-based inhibitory motif (ITIM) and an immunoglobulin tyrosine tail (ITT)-like domain, while CD226 has an ITT-like domain and CD155 has an ITIM. CD155 has a higher affinity for TIGIT than other ligands and is an immune-suppressing receptor like TIGIT. CD226 is an immune-activating receptor whose ligand is CD155, but it can be competed for by TIGIT. Additionally, TIGIT inhibits CD226 by disrupting its dimerization. Fap2 is a special protein ligand for TIGIT, which is secreted by *F. nucleatum* and can cause suppression of immune cells. Created in https://BioRender.com.

The extracellular structural domains of TIGIT share homology with other protein receptor members of the family, such as CD155, CD96, CD226 and nectin-4 (PVRL4) [21]. The main ligands for TIGIT are CD155 (PVR), CD112 (PVRL2, nectin-2), CD113 (PVRL3, nectin-3) and nectin-4 (PVRL4) [21, 25, 43]. The interaction between TIGIT and its ligands is shown in Fig. 1. Different ligands have different affinities for TIGIT, with CD155 having the highest affinity. CD226 is another important immune-activating receptor that competes with TIGIT for the ligand CD155, but it has much lower affinity. In addition to this, other ligands of TIGIT may also be involved in interactions, forming a complex immunoregulatory network, which should be considered holistically in immunotherapy. For example, TIGIT–CD112 axis has recently been identified as an important factor that influences immunity against tumor in neuroblastoma [44], although CD112 has a higher affinity for CD112R and has been considered as another axis in immunotherapy [22, 23]. Nectin-4 is currently considered to be an exclusive ligand for TIGIT, as no other members of the PVR-like family have been found to bind to it [45]. The Fap2 protein secreted by *F. nucleatum*, is a special TIGIT ligand; it can directly interact with human TIGIT and cause NK cell inhibition [46].

TIGIT gene is located on q13.31 of chromosome 3 in *Homo sapiens*. The mRNA sequence length of TIGIT transcript is 2926 nucleotides, while its coding sequence (CDS) length is 732 bp. A TIGIT molecule consists of 244 amino acids divided into three main regions or domains: the extracellular IgV region with 141 amino acids, the transmembrane domain with 23 amino acids, and the cytoplasmic tail with 80 amino acids [16, 47–50]. The cytoplasmic tail contains an immunoreceptor tail-tyrosine (ITT)-like motif and an immunoreceptor tyrosine-based inhibitory (ITIM) motif, which are conserved in humans and mice as they are critical for mediating inhibitory signaling [48, 50]. Work by Stengel et al. [47] in 2012 has helped to resolve the structure of the TIGIT/PVR complex and suggested that it is a heterotetramer consisting of CD155-TIGIT-TIGIT-CD155, where characteristic lock-and-key structures were evident. TIGIT’s Y113 and CD155’s F128 constitute the ‘key’ of the structure, inserting inside the PVR family conservative AX6G ‘lock’ of each other. According to this model, dimers of TIGIT and CD155 tend to be interspersed and bound; each paired with a neighboring ligand for the formation of the signaling cluster [47].

### TIGIT inhibitory signals

#### Inhibitory signaling by TIGIT cytoplasmic tails

When bound to a ligand, TIGIT can directly inhibit human NK cytotoxicity in an ITIM motif-dependent manner. This was demonstrated by generating the amino acid 231-truncated TIGIT molecule [51]. It is worth noting that direct suppression of immune function by TIGIT cytoplasmic tails appears to be present only in NK cells and is not observed in T cells [50]. Most studies addressing direct intracellular signaling by TIGIT were performed using the YTS/TIGIT cell line of human NK cells [21]. Direct inhibitory signaling of TIGIT has also been observed in mice, and this signaling can be accomplished by ITIM or ITT-like motif alone [48]. The ITT-like motif is also important for inhibiting human NK cytotoxicity, as its phosphorylation causes the recruitment of growth factor receptor-binding protein 2 (Grb2), which then recruits SH2-containing inositol phosphatase-1 (SHIP-1), leading to inhibition of P13K and MAPK signaling [52]. Another study showed that ITT-like motif recruits β-arrestin 2, leading to IFN-γ inhibition [53]. TIGIT was also reported to inhibit the phosphorylation of ERK1/2 and ZAP70/Syk in response to CD155 stimulation, thereby suppressing the NK cell function [54].

#### CD155-mediated inhibitory signaling

CD155 can be expressed by many normal human cell types, but it is upregulated in a variety of tumors, including non-small cell lung cancer (NSCLC), melanoma, colorectal cancer and glioblastoma [55–57]. It contains an ITIM motif, suggesting the possibility of signaling to the cell interior. Dendritic cells (DCs), as antigen-presenting cells (APCs), express CD155 and can hence engage TIGIT on T or NK cells. DCs can therefore receive signals via their CD155 that upregulate the synthesis of immunosuppressive cytokines (IL-10) and downregulate pro-inflammatory cytokines (IL-12); this diverts DC function towards immuno-tolerance [50]. The interplay between TIGIT and CD155 on APCs can empower TIGIT to indirectly inhibit T and NK cytotoxicity by inducing the anti-inflammatory or immune-tolerance phenotype in APCs. This is further supported by the observation that TIGIT-Fc fusion protein induces CD155-expressing macrophages to upregulate the production of IL-10 and agonistic anti-TIGIT antibody induces upregulation of several TFs, chemokine receptors and Treg effector molecules, including IL-10 or Fgl2 in Tregs, re-coding them to a more inhibitory phenotype [58].

#### CD226-dependent immune inhibition

In addition to binding to CD155, TIGIT can inhibit immune responses by competing for ligands with the immune-activating receptor CD226. CD226 has been shown to play an important role in cell contact and TCR signaling [59] and to promote T cell activation [60]. TIGIT binds CD155 with higher affinity, implying that there is a competitive relationship between TIGIT and CD226 in the case of co-expression and that TIGIT preferentially exerts an immunosuppressive effect [22]. Blocking of CD226 was reported to eliminate CD4^+^ T cell functional activation by TIGIT knockdown [61]. Besides its ability to compete with CD226 for the ligands CD155 and CD122, TIGIT was also reported to inhibit CD226 signaling. Specifically, the result of fluorescence resonance energy transfer demonstrated that TIGIT disrupts the dimerization of CD226 on T cell surface, and thereby inhibiting its ability to bind to CD155 [30].

#### TIGIT suppresses TCR-driven activation signals

Although TIGIT does not seem to signal to T cells through its cytoplasmic tail, it can still suppress T cell activation through an intracellular mechanism. This is based on the observation that TCR-driven activation signals can be inhibited by TIGIT-mediated signaling [61, 62]. Moreover, TIGIT was reported to downregulate the expression of several genes that are associated with TCR and T-cell activation [62]. Together, these observations suggest that TIGIT delivers inhibitory signals to T cells through specific yet-to-be-identified mechanisms that seem to operate independently of extracellular triggers or CD226 competition.

#### TIGIT promotes Treg polarization to an immunosuppressive phenotype

In addition to CD4, CD8, and NK cells, Tregs also express TIGIT. Demethylation of the TIGIT locus and FOXP3 binding was reported to upregulate TIGIT expression on Tregs, whereas methylation of the TIGIT gene locus can restrict FOXP3 binding. Increased expression of TIGIT on Tregs seems to enhance their immune inhibitory phenotype [21, 23]. Several gene signatures (FOXP3, Helios, neuropilin-1, CTLA-4, PD-1 and LAG-3) are also found to be upregulated in TIGIT^+^ Tregs within the tumor microenvironment (TME), turning Tregs to a more immunosuppressive phenotype [23]. The cell types inhibited by TIGIT-expressing Tregs demonstrate specificity; in particular, they inhibit only T helper 1 (Th1) and Th17 cells, but not the Th2 cells [41].

## Co-inhibition of TIGIT along with other ICs

**Fig. 2:**
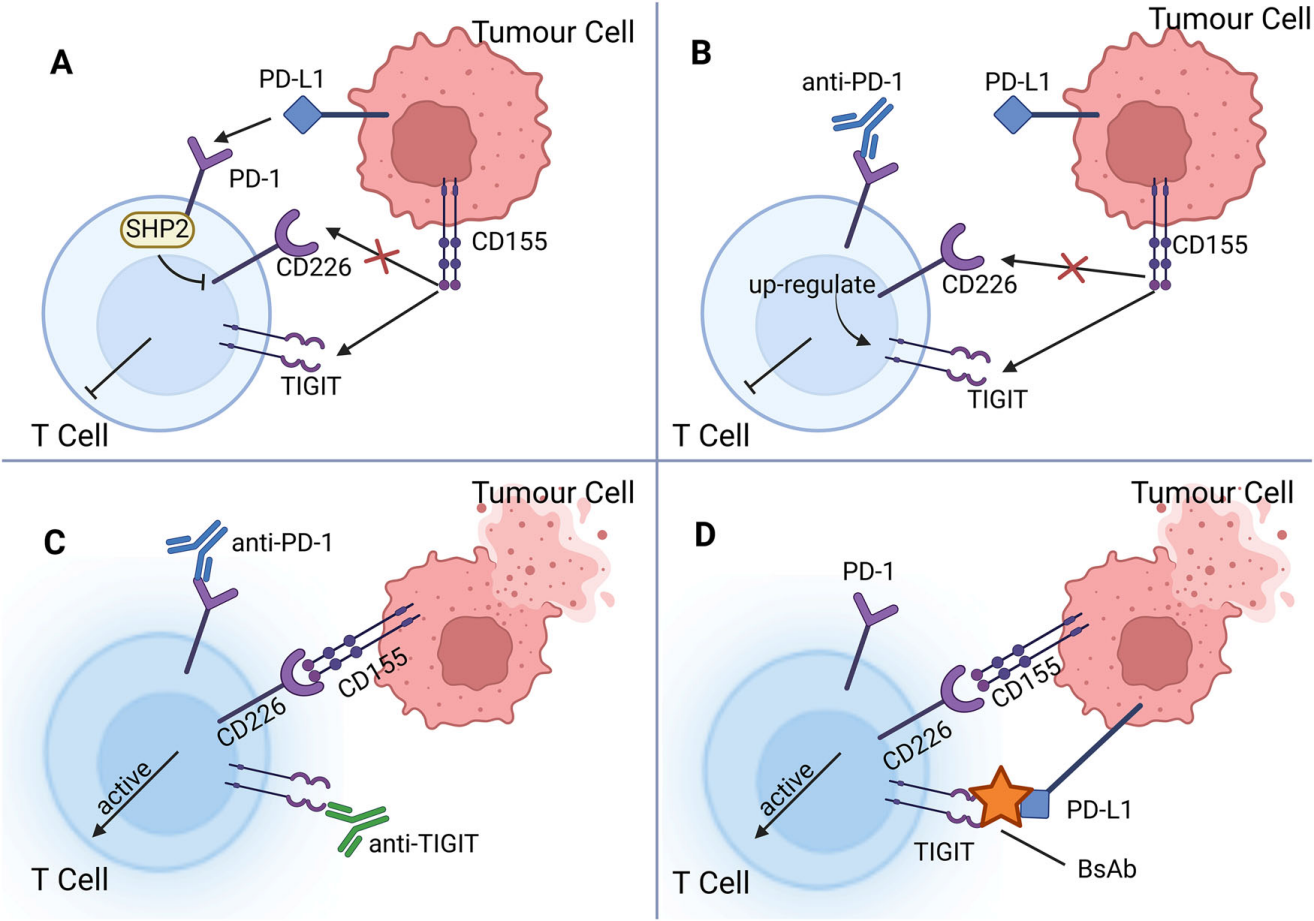
Synergistic effects of TIGIT and PD-1 immune checkpoints and co-inhibition therapy. **A** Both TIGIT and PD-1 inhibit signaling from the immune-activating receptor CD226. PD-1 binds to PD-L1 and recruits SHP2, ultimately leading to the inhibition of intracellular signaling from CD226. TIGIT accomplishes this extracellularly by competing with CD226 and disrupting the structure of the CD226 dimer. **B** Using antibodies to inhibit the PD-1/PD-L1 pathway increases TIGIT expression, leading to immune inhibition. **C** Combination of anti-TIGIT and anti-PD-1 antibodies can release the inhibition of CD226 signaling and activate T cells. **D** Bispecific antibodies (bsAbs) inhibit both TIGIT and PD-1/PD-L1 and bridge immune cells and tumor cells. Created in https://BioRender.com.

As discussed earlier, several anti-TIGIT mAbs have been developed to inhibit TIGIT. Unfortunately, clinical trials using those mAbs in solid tumors yielded poor outcomes compared to pre-clinical models, and the ORR was close to null [22, 63, 64]. One possible reason for unsatisfactory efficacy is redundancy in the TIGIT signaling pathway. CD155, as the ligand of TIGIT, has diverse signaling pathways, such as transmitting inhibitory signals via CD96 or signals to DCs via its own cytoplasmic tail. Another reason is the multiple immunosuppressive mechanisms in the TME, including compensation between ICs, infiltration of Tregs and the synergistic mechanism with PD-1 that will be addressed in this review later [22, 63, 64]. TIGIT/CD155 axis and PD-1/PD-L1 axis exhibit a synergistic effect at the molecular level, whereby they both inhibit the CD226 signaling pathway. Figure 2 shows the phenomenon of synergy and co-expression of the two axes and the advantages of co-blockade therapy, which has the potential to overcome the low response rate to anti-TIGIT monotherapy. In particular, PD-1 and TIGIT are usually co-expressed on the cell surface and blocking PD-1 upregulates TIGIT. This may be a contributing factor to the unsatisfactory outcomes of anti-PD-1 therapy [65]. Several clinical trials have confirmed that, despite the increase in TAREs, combination therapy with anti-TIGIT and anti-PD-1 demonstrated better efficacy than using either mAb alone [66, 67]. At the molecular level, PD-1 is surprisingly identified to inhibit the phosphorylation of CD226 through its binding to PD-L1 and the subsequent recruitment of SHP2, which inhibits CD226 immune activation signaling intracellularly [65]. This reveals a synergistic mechanism whereby TIGIT competes for CD226 signaling extracellularly, while PD-1 acts intracellularly. To fully restore CD226 function, both ICs need to be blocked simultaneously. These observations have provided the rationale for a therapeutic approach involving the targeting of both TIGIT and PD-1. The function of the Fc segment of the antibody in TIGIT and PD-1 co-inhibition therapy was recently demonstrated. The Fc segment of tiragolumab probably binds Fcγ receptors (FcγR) on myeloid cells in tumors, which can cause inflammation of macrophages, monocytes and DCs. Meanwhile, researchers also observed down-regulation of immunosuppressive genes in tumor-associated Tregs after Fc-active anti-TIGIT antibody administration, facilitating the efficacy of combination therapy. This suggests that Fc function of anti-TIGIT antibodies and myeloid activation are important for TIGIT/PD-1 co-inhibition therapy [68].

The anti-tumor efficacy of co-targeting TIGIT and PD-1/PD-L1 using individual mAbs against these ICs has been demonstrated by several trials. Compared to single-antibody use, co-blockade therapy has significant advantages in restoring T-cell viability and overall survival [30, 69–71]. Based on the theoretical basis of co-inhibition and improved clinical outcomes, the use of bispecific antibodies (bsAbs) recognizing TIGIT and PD-1 was also tested. In this case, linkers were used to connect anti-PD-L1 IgG (YN035) and anti-TIGIT heavy-chain-only antibodies (hTIGI7.6 and hTIGI7.11E) to generate the anti-PD-L1/TIGIT bsAb. As the hTIGI7.11E has an affinity for human TIGIT only, the investigators tested its anti-tumor effect in the MC38 tumor-bearing model of TIGIT humanized mice. The result demonstrated a significant increase in immune cell (mainly cytotoxic T cell) infiltration, accompanied by tumor suppression, as well as a reduction of Tregs in the TME. In addition to the blocking effect of the two monospecific antibodies, bsAbs can connect immune cells to tumor cells and enhance cross-talk between TIGIT^+^ T cells and PD-L1^+^FcRs^+^ DCs, which further promoted anti-tumor activity [72]. However, the therapeutic utility of additional co-inhibition strategies involving TIGIT and other IC targets has yet to be fully explored. Previous studies have demonstrated that the T-cell immunoglobulin and mucin domain 3 (TIM-3) and TIGIT are co-expressed on NK cells from hepatitis B virus-related hepatocellular carcinoma (HBV-HCC) patients and their co-expression associates with T cell depletion, attenuated cytotoxicity and strongly correlates with disease progression [35]. Based on this, a PD-L1×TIGIT×LAG3 tri-specific antibody has been developed and has been shown to be superior to the benchmark antibody combinations [73].

In addition, TIGIT immunosuppressive mechanism is reminiscent of CTLA-4, as they both compete for ligands with immune-activating receptors. CTLA-4 on T cells competes with CD28 by binding to B7 molecules (CD80/CD86) on APCs [74]. As the earliest discovered ICs, combined inhibition of CTLA-4 and PD-(L)1 has also been explored in recent years. Several preclinical trials have demonstrated that the combination of PD-1/PD-L1 and CTLA-4 inhibitors has a significant synergistic effect in reversing cytotoxic CD8^+^ T cell exhaustion, attenuating the tumor load and prolonging survival [75]. A number of Phase II and Phase III clinical trials are also ongoing, with data showing some median OS improvement in the PD-(L)1/CTLA-4 combination therapy group; however, the addition of grade 3-4 TAREs raises concerns about safety [75]. Identifying which IC to co-target with PD-1 for synergistic therapeutic enhancement remains an open and intriguing question. While TIGIT and CTLA-4 share overlapping immunosuppressive mechanisms, TIGIT engages a broader array of inhibitory pathways. This expanded complexity necessitates careful clinical evaluation, particularly since the dominant pathway mediating antitumor efficacy remains undefined. CTLA-4 primarily acts during the initial activation phase of T cells in lymphoid organs, whereas PD-1 blockade occurs on T-cell effector phase and can activate TCR-mediated effector functions [22, 75]. This suggests that co-blockade of PD-1 and CTLA-4 facilitates the entire process of T cells from activation to exercise of effector functions, whereas co-blockade of PD-1 and TIGIT provides enhanced tumor killing by activated T cells. In terms of efficacy and safety, as approved therapies, PD-1/CTLA-4 co-blockade showed significantly prolonged survival, but with more severe TAREs (mainly influenced by the dose of CTLA-4 inhibitors); while PD-1/TIGIT co-blockade has milder side effects, but clinical trials may show varying response rates that need to be supported by further data [23, 75]. In addition, some biomarkers are under validation for PD-1/CTLA-4 co-inhibition, such as high PD-1 expression and high TMB, suggest a narrower scope (melanoma and renal cell carcinoma) of applicability for this therapy after taking safety considerations into account [76]. PD-1/TIGIT co-inhibition is more broadly applicable, but it should be considered with caution in screening patients due to the lack of validated biomarkers [64].

## Small molecule inhibitors targeting TIGIT

Low tissue penetration of mAbs as drugs [13, 77] coupled with poor drug utilization [78] are major limitations of the utility of mAbs in cancer immunotherapy. One way to address this issue is the employment of small molecule inhibitors (SMIs) to block ICs like TIGIT and PD-1. Unlike mAbs, SMIs have a much smaller spatial resistance and are immunogenic; that is why they do not elicit immune responses. They also have the added advantages of low cost, ease of transport and preservation, well-controlled risk, and amenability to modifications aiming to increase affinity and stability [13, 79, 80]. Several principles can be applied in designing SMIs targeting ICs like TIGIT: binding blockade, transcriptional inhibition, and promotion of degradation [13], which offers flexibility in action mode compared with mAbs. Table 1 summarizes the different strategies for blocking TIGIT.

**Table 1:** Different strategies for targeting TIGIT.

Type of agent	Inhibitor name	Developer	R&D phase	Mechanism of action	Combination drug	Indications
Monoclonal antibody	Tiragolumab	Genentech	Phase III clinical trial	Blocking TIGIT binding to ligand	Atezolizumab	Lung/oesophageal cancer
	Vibostolimab	Merck	Phase III clinical trial		Pembrolizumab	PD-1-resistant solid tumors
	Domvanalimab	Gilead	Phase III clinical trial		Zimberelimab	Stomach/lung cancer
	Ociperlimab	BeiGene	Phase III clinical trial		Tislelizumab	Cervical/lung cancer
	Etigilimab	Mereo BioPharma	Phase II clinical trial		Nivolumab	Solid tumors
Bispecific antibody	BiPT-23	Oricell Therapeutics	Preclinical research	Targeting TIGIT and PD-L1	None	Undisclosed
	SHR-2002	Jiangsu Hengrui Pharmaceuticals	Preclinical research	Targeting TIGIT and CD155	None	
	Rilvegostomig	AstraZeneca	Phase II clinical trial	Targeting TIGIT and PD-1	None	
	IBI-321	Innovent	Phase I clinical trial		None	
Trispecific antibody	GB266T	Ab Therapeutics	Preclinical research	Targeting PD-L1/TIGIT/LAG-3	None	
Chemical compound	Elraglusib (9-ING-41)	Shaw et al.	Preclinical research	GSK3 inhibitor (controversial)	anti-PD-(L)1	
	Gln (TrT)	Li et al.	Preclinical research	Inhibits the TIGIT signaling pathway with high penetration	anti-PD-(L)1	
	Hemin	Zhou et al.	Preclinical research		undisclosed	
Peptide-based inhibitor	DTBP-3	Zhou et al.	Preclinical research	Protein-peptide interactions	nivolumab (anti-PD-1)	
RNA	TIGIT-Fc-LIGHT and SIRPα-Fc-CD40L	Shattuck Labs	Preclinical research	Producing a hexameric fusion protein	anti-PD-L1 (clone 10F.9G2)	
	miR-486-5p, etc.	Assal et al.	Preclinical research	Knockdown of lncRNAs	None	

It is well established that the anti-tumor activity of elraglusib (9-ING-41) involves the downregulation of the expression of several IC molecules, including TIGIT, and inhibition of glycogen synthase kinase-3 (GSK-3) [81]. As an ATP-competitive SMI of GSK-3, elraglusib has been reported to exert suppressive anti-growth effects in a variety of cancers [82–84]. GSK-3β phosphorylates a variety of proteins involved in NF-κB and c-Myc signaling, which enable it to modulate immune escape and regulate oncogenesis, cell cycling, and apoptosis. Binding of the co-stimulatory receptor CD28 and TCR induces phosphorylation of GSK-3β, leading to its inactivation [85–88]. Co-culturing of antigen-specific CD8^+^ CTLs with EL4 lymphoma cells showed that elraglusib significantly increased lactate dehydrogenase release, suggesting increased target lysis [81]. Whether alone or in combination with anti-PD-1, elraglusib inhibited the growth of melanomas in a B16 mouse model. When used in combination with anti-PD-1, elraglusib was most effective when administered to hosts that had already received anti-PD-1 mAb immunotherapy [81]. The anti-tumor effect is associated with down-regulation of mRNA relating to several IC molecules, including TIGIT, PD-1 and LAG-3 [81]. The mechanism is hypothesized to be similar to that in GSK-3 inhibition, which enhances Tbet (Tbx21) transcription and thereby represses promoter activity in target genes [89, 90].

Gln (TrT) is another small molecule that was reported to bind both human and mouse TIGIT and PD-1 molecules. Molecular docking suggested that Gln (TrT) interacts with residues on the interaction surface of TIGIT/PVR and PD-1/PD-L1. Cell-based analysis showed that Gln (TrT) can effectively block TIGIT and PD-1 binding to their ligands and restore IL-2 secretion by Jurkat cells [79]. In the MC38 mouse model, Gln (TrT) increased CD8^+^ T-cell infiltration in the TME and promoted infiltrating cells to secrete IFN-γ at tumor sites, draining lymph nodes, and spleen; mice treated with this SMI showed significant tumor growth inhibition. Immunohistochemical analysis of tumor tissues similarly demonstrated that Gln (TrT) inhibits tumor cell proliferation and promotes apoptosis [79]. Hemin, another small molecule that can interact with TIGIT/PVR, was developed based on virtual molecular docking and cell-based closure assays, and can interact with TIGIT/PVR and enhance anti-tumor CD8^+^ T cell killing activity. It acts synergistically with IFN-γ to induce ferroptosis. A combination of hemin plus anti-PD-1/PD-L1 mAb treatment rescued anti-PD-1 resistance in a B16 resistance melanoma cell model [91].

In addition to small chemical compounds, peptides are also developed for IC blockade. The advantage of peptides over other small molecules is that they have more similar properties to proteins, allowing them to bind to the flat hydrophobic surface of proteins. DTBP-3 (GGYTFHWHRLNP), a peptide-based ICI targeting TIGIT, was developed by mirror phage display technology to solve the problem of excessive degradation of L-peptide in mice and human serum. In vitro and in vivo testing showed that this peptide increases IFN-γ secretion and inhibits tumor growth in a CD8^+^ T-cell-dependent manner. Encouragingly, ^D^TBP-3 has shown additional advantages in the treatment of a PD-1-resistant model, because it can inhibit tumor growth and metastasis [92].

As of now, few SMIs have been developed to target the TIGIT/PVR axis; this is probably due to difficulties in screening and the limited binding affinity of small molecules to protein interaction sites. While there are many advantages to SMIs, the potential for off-target toxicity and diminished effectiveness due to short half-life in vivo are worth considering [93]. Recently, researchers engineered lipid nanoparticle (LNP)-encapsulated mRNAs that encode TIGIT-Fc-LIGHT, a complex hexameric fusion protein linking the TIGIT blocker and TNFL agonist. In vivo administration resulted in sustained hexameric protein production, with increased tumor antigen-specific cytotoxic T cells observed as well as extended survival [94]. The use of encapsulated mRNA rather than the protein itself reduces the risk of immunogenicity with repeated dosing and is more cost-effective and favorable for large-scale dissemination. LNPs provide protection for the mRNA cargo and extend the in vivo half-life of the drug. Another study utilized an epigenetic strategy using non-coding RNAs (ncRNAs) to dually target the CD155/TIGIT and PD-1/PD-L1 axes, and inhibition of PD-1/PD-L1 and TIGIT/CD155 expression was observed in HCC, resulting in enhanced cellular toxicity [95]. This offers a means of targeting ICs such as TIGIT that is distinct from blockade, namely, inhibition at the gene expression level.

## TIGIT in CAR-T cells

**Fig. 3:**
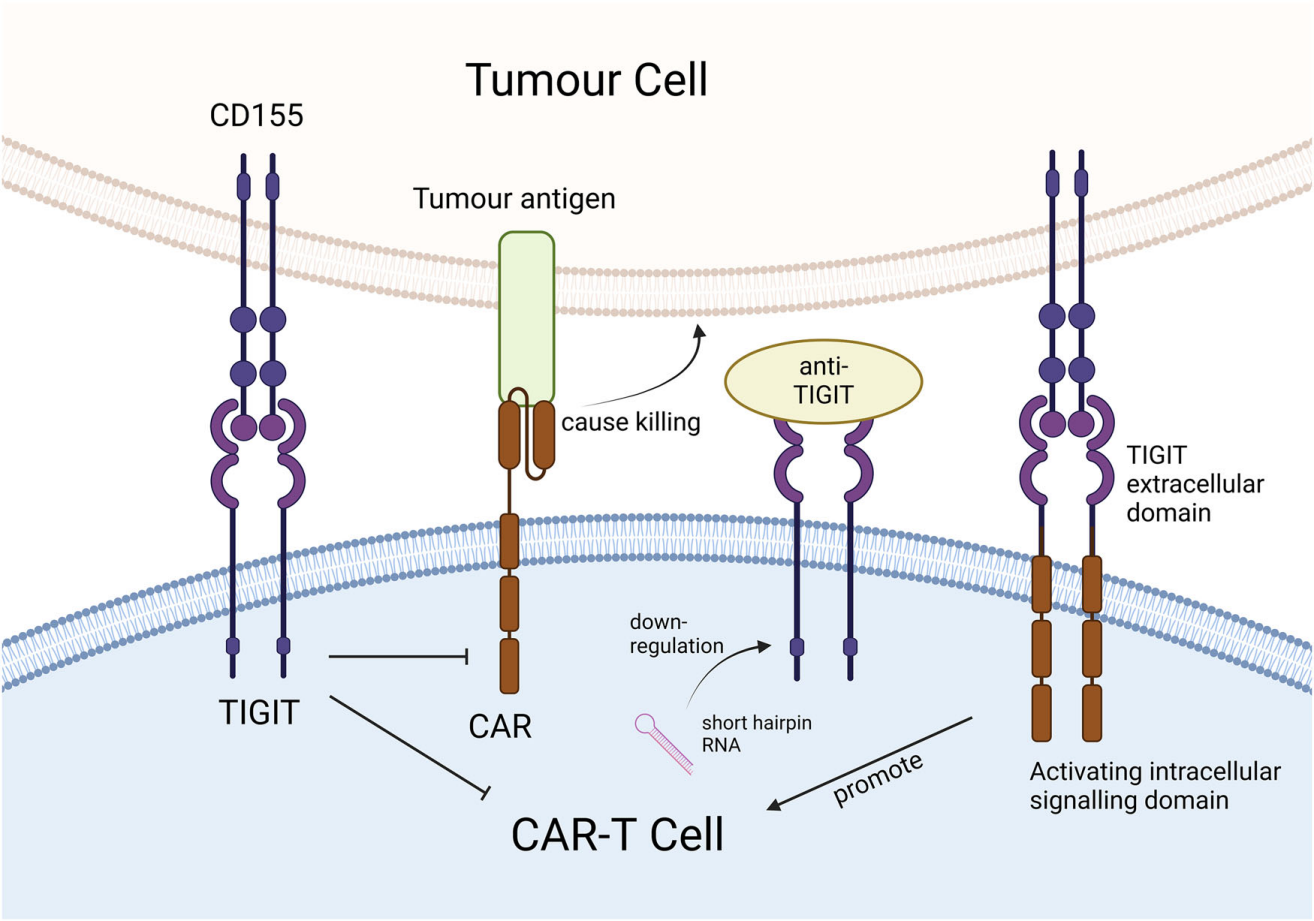
Blocking TIGIT in CAR-T therapy. CAR on the surface of genetically engineered T cells recognize tumor antigens and induce killing, but binding of TIGIT to CD155 inhibits this effect. Blocking of TIGIT by antibodies or its down-regulation by shRNA can be favorable to activation of CAR-T cell immune function. The combination of TIGIT extracellular domain and activating intracellular domain can promote tumor killing. Created in https://BioRender.com.

CAR-T cell therapy is based on the construction of chimeric T cell antigen receptors. This approach has proven effective in treating a wide range of hematological malignancies [96]. CAR-T cells express artificially modified chimeric antigen receptors, which are composed of a single-chain variable fragment (ScFv), a hinge region, a transmembrane domain, and a co-stimulatory and activation domain. ScFvs are designed to recognize a specific epitope on a tumor antigen, allowing the CAR-T cells to specifically target tumor cells overexpressing the antigen. Co-stimulatory and activation domains assume the role of intracellular signal transduction that leads to CAR-T proliferation and activation [97, 98]. However, the application of CAR-T therapies is of limited utility against solid tumors due to depletion of CAR-T cells in the TME [99]. Figure 3 describes the effects of TIGIT on CAR-T cells and methods to block TIGIT in CAR-T therapy. Interestingly, high TIGIT expression is a biomarker of non-proliferative, highly differentiated and depleted CAR-T cells; therefore, blocking of TIGIT may restore CAR-T cell function [100]. This is further supported by the observation that engagement of TIGIT suppresses CAR-T cells; this limits CAR-T’s immunotherapeutic utility and contributes to recurrence [101]. These data suggest that TIGIT inhibition can be combined with CAR-T therapy for better efficacy.

Blocking or downregulating TIGIT expression in CAR-T cells was assessed to overcome the TIGIT-hindering effect on CAR-T immunotherapy. Treatment of CAR-T cells targeting mesothelin (MSLN) with α-TIGIT antibodies, was reported to block TIGIT and enhance MSLN CAR-T cell killing activity [97]. Following this trail, researchers designed a new CAR structure that contains multiple spacers and CD28 co-stimulatory structural domains, followed by the use of secretory anti-α-TIGIT scFv. The cell was named MT CAR-T and has the ability to express anti-α-TIGIT scFv, which blocks TIGIT on the surface of T cells by self-delivery. In vitro and in vivo studies demonstrated that MT CAR-T cells can block their own TIGIT, allowing for CAR-T cell activity to be optimized. The efficacy of this approach was superior to that of anti-TIGIT antibodies plus MSLN CAR-T cells. Noteworthy mentioning, while mice treated with MSLN CAR-T cells showed recurrence, no recurrence was observed in MT CAR-T cell-treated mice [97]. Approaches to downregulate TIGIT in CAR-T cells were also developed and tested. For example, CAR-T cells targeting CD19 were developed using a dual short hairpin RNA cassette integrated into the CAR vector. The results showed simultaneous down-regulation of PD-1 and TIGIT along with increased immunoreactivity of such CAR-T cells. This outcome was shown to be CD226-dependent. It is worth noting that upregulation of CD112 and CD155 was observed in such CAR-T cells, which may contribute to CD226-stimulating signaling between adjacent cells. It is interesting to note that downregulation of TIGIT altered the transcriptomic profile of CAR-T cells, with reduced expression of the inhibitory receptors LAG3 and CD244 and the chemokine genes XCL1, XCL2 and CCL3 and increased expression of naive/central memory phenotypes (IL7R, BCL6, and CD27) and active glucose metabolism genes (HK2, PFKFB4, and PDK1). These observations suggest that TIGIT downregulation may help to slow or hinder CAR-T cell depletion [102].

Use of chimeric co-stimulatory switch receptors to turn inhibitory receptor-delivered signals into stimulatory ones is another approach to engaging TIGIT as an immunotherapeutic modality in cancer. CD155 is overexpressed in many cancers, which can be utilized by this approach to target and kill tumor cells through the fusion of TIGIT’s extracellular domain (ECD) with intracellular stimulatory domains. TIGIT’s ECD and CD28’s cytoplasmic tail were linked using transmembrane (TM) portions derived from TIGIT or CD28 to generate chimeric receptors. The reprogrammed CAR-T cells were proven to have enhanced T-cell function and cytokine secretion, as well as superior anti-tumor cytotoxicity in a mouse model. Enhanced function of CAR-T cells was associated with CD155 expression in target cells, and no increase in TNFα secretion was detected when co-cultured with CD155^-^ targets. Intracellular signaling is mediated by CD28 because its signaling cascade molecules pERK and Bcl-xL are increased [103]. This suggests that the CAR successfully converted the inhibitory signals of TIGIT into activating signals. Similarly, chimeric IC switching receptors (CISRs) can be designed for the association of multiple IC extracellular structural domains. Another study designed PD1-TIGIT CISR, which combines the extracellular domain (ECD) of PD-1/TIGIT and the T cell co-stimulatory molecule CD28 ICD, leading to intracellular positive signal activation when binding to corresponding IC ligands. T cells expressing PD1-TIGIT CISR showed enhanced proliferation and anti-tumor activity [104]. It is worth emphasizing that, with the exception of one ongoing clinical trial (NCT04836507), clinical validation is still lacking for the majority of these modalities.

## Dilemmas in clinical trials of TIGIT blockade

Roche recently disclosed complete data from the failed phase III clinical trial SKYSCRAPER-01, in which the anti-TIGIT drug tiragolumab, in combination with the PD-1 inhibitor atezolizumab, was used in patients with NSCLC. The data showed that the median progression-free survival (PFS) and OS did not meet the primary endpoints. As assessed by the investigators, the median PFS in the tira + atezo group was 7.0 months compared to 5.6 months in the pbo + atezo group (data cut-off Mar 2022; HR 0.78; 95% CI 0.63, 0.97; *p* = 0.02), while the median OS in the tira + atezo group was 23.1 months compared to 16.9 months in the pbo + atezo group (data cutoff Sep 2024; HR 0.87; 95% CI 0.71, 1.08; *p* = 0.22). Although the tira + atezo group showed some advantages in PFS and OS, this difference did not reach the pre-specified statistical significance threshold (P<0.01) and the increased risk ratio created concern [105]. As the industry’s benchmark, Roche’s tiragolumab phase III clinical failure will undoubtedly dampen the market’s confidence in the TIGIT target. On the other hand, Bristol Myers Squibb, Merck and BeiGene terminated the phase II/III clinical trials of their own anti-TIGIT mAbs due to unsatisfactory data, which have aggravated this concern [106–108]. GSK has recently announced the termination of the development of TIGIT mAb belrestotug after it failed to meet clinical significance criteria for PFS in the GALAXIES Lung-201 clinical trial. However, PD-1 expression level (>1% or >50%) has become almost the sole criterion for patient screening, as this is due to the absence of effective biomarkers in anti-TIGIT therapy.

The expectation of TIGIT is not only to enhance the efficacy of patients responding to anti-PD-1, but also to improve the prognosis of PD-1-resistant patients through combination therapy, which means that biomarker discovery is crucial. Meanwhile, this can help to better screen patients in clinical trials and immunotherapy. In addition, despite multiple failures in Phase III clinical trials in different therapeutic areas, some Phase II/III clinical results still support TIGIT as a promising target. ARC-7 is a phase II clinical trial that evaluated the efficacy of anti-TIGIT mAb (domvanalimab) + anti-PD-1 mAb (zimberelimab), domvanalimab + zimberelimab + etrumadenant or zimberelimab alone in NSCLC. Results showed that both groups containing domvanalimab demonstrated sustained ORR improvement (41% and 40% vs. 27%), and a significant increase in PFS (12 and 10.9 vs. 5.4 months) [109]. In addition, studies such as GALAXIES-201 and EDGE-Gastric have observed positive preliminary efficacy data [64]. Roche’s tiragolumab also has a role in the treatment of esophageal cancer, with the phase III clinical SKYSCRAPER-08 showing a strong superiority of the combination over chemotherapy [110]. However, it has been widely questioned for not setting an anti-PD-1 monotherapy control. In response, Roche opened SKYSCRAPER-07, combining tiragolumab with tecentriq (atezolizumab, anti-PD-L1), and comparing it to tecentriq monotherapy, but no data are currently available [111].

Interestingly, of the current major anti-TIGIT mAbs, only domvanalimab is Fc-inactivated, and the rest have an active Fc part. The prevailing view is that active Fc segments can contribute to the efficacy of TIGIT mAbs in immunotherapy through the action of FcγR [112], but more clinical validation is needed. Further efforts are needed to develop biomarkers for TIGIT to screen for potential responsive patients to anti-TIGIT/PD-1 combination therapy. Clinical trials by Roche and Arcus suggest that anti-TIGIT therapies may be effective for specific cancer types. In the future, when screening for responsive patients becomes a reality, the TIGIT target may play an important role in precision medicine. This assertion is further substantiated by the observation that despite the data from the phase III trials failing to attain the primary endpoint, enhancements in PFS and OS were nevertheless obvious [105]. It is important to note that different anti-PD-1 mAbs were used in clinical trials, but it cannot be ignored that Keytruda (pembrolizumab) is the one that demonstrates superiority in efficacy. A conclusive demonstration of the combination therapy’s efficacy requires statistically superior clinical outcomes compared to pembrolizumab monotherapy.

## Discussion

Recently, unsatisfactory results from several phase III clinical trials have hampered the clinical translation of TIGIT as a target, with giants such as Roche and BeiGene announcing their withdrawal from their development programs for TIGIT mAbs. The reasons for the failure could be multiple. First, the lack of biomarkers of anti-TIGIT therapy brings difficulties in measuring its efficacy. Current anti-TIGIT/PD-1 clinical trials use PD-1 expression as a single criterion, which does not consider the reaction on the TIGIT/PVR axis. Second, the controversy over the biological mechanism. Unlike anti-PD-1 mAbs, most of the antibodies developed against TIGIT are Fc-active [22]. This is based on preclinical observations that antibodies with effector functional Fc preferentially deplete Tregs in vitro and in vivo [112, 113]. Recently, a study analyzed data from the clinical trial CITYSCAPE demonstrate that the functional Fc segment of tiragolumab facilitates anti-TIGIT immunotherapy in an FcγR-dependent manner on intratumoral macrophages, monocytes, and Tregs, supporting this view [68]. However, the opposing view states that Fc-active antibodies may impair extensive TILs due to the widespread expression of TIGIT on T/NK cells [112]. It is a possible direction to optimize the Fc form to allow the mAbs to deplete Tregs in the TME, without affecting effector T cells. Third, the issue of experimental design. The phase II clinical CITYSCAPE showed bright results, while the subsequent phase III trials SKYSCRAPER-01 and SKYSCRAPER-02 were both declared as failures [24], exposing problems in patient selection. In both phase III clinical trials, no information was available on CD155 expression in patients [105, 114]. One question is whether there is a synergistic effect of PD-L1 and CD155 expression in NSCLC and SCLC? It has been shown that the inhibitory effect of TIGIT on CD8^+^ T cells is mainly due to competition with CD226 rather than other mechanisms [65]. This suggests that in CD8^+^ T cells, promoting CD155 and CD226 interactions is the ultimate goal. Of course, as mentioned earlier, the Fc segment of tiragolumab is activated to deplete infiltrating Tregs, but a more statistically rigorous design should be considered. In addition, data from several phase III clinical trials support a role for TIGIT in other cancers [24], and blocking TIGIT in non-advanced tumors can be attempted. Precise patient grouping considering the above criteria could be possible in the future, as well as developing optimized antibodies such as Fc-designed mAbs and bsAbs.

Novel strategies of targeting TIGIT bring new hope to this target. BsAbs not only achieved co-blockade of TIGIT and PD-1, but also bridged the gap between immune or tumor cells, which will further facilitate the immune response. SMIs can circumvent the disadvantages of poor tissue penetration and low utilization of antibodies. Several small molecules have been designed to target TIGIT, but there are still few studies in this area, and there is a lack of clinical data. The penetration capability of SMIs may bring unique advantages in immunotherapy, particularly in the case of anti-TIGIT, which involves multiple immune cell types. Once the efficacy of targeting TIGIT is better demonstrated, the anti-TIGIT SMIs will become promising alternatives to mAbs. Because of the limited efficacy of blocking TIGIT alone, these approaches to targeting TIGIT should be flexibly combined with other immunotherapies, such as anti-PD-1/LAG-3 or cancer vaccines [115]. Furthermore, TIGIT has been proven to be an influential factor in the poor efficacy of CAR-T therapy. Blocking TIGIT or associating it with activating intracellular signaling domains has emerged as a promising approach to improve CAR-T therapy.

When targeting TIGIT in immunotherapy, the impact of blockade or activation should be considered on a larger scale than the TIGIT/PVR/CD226 axis, as there are many shared ligands in this signaling pathway. For example, CD112 is one of the ligands of TIGIT but mainly binds to CD112R, and the CD112/CD112R axis has been suggested as a target for immunotherapy [44]. CD155 was investigated as the primary ligand for TIGIT in some early phase I/II clinical trials [116]. A TIGIT/CD155 bsAb has also been developed, indicating the potential of targeting this protein [117]. However, blocking CD155 requires consideration. Its widespread expression in human immune cells, epithelial cells and endothelial cells may cause unpredictable safety risks, and maintaining activation of the CD226 signaling pathway is another consideration [56]. CD155 expression in tumor tissue, as well as TIGIT expression in peripheral blood/TILs, TMB and TME immunological profile can be considered when selecting biomarkers. Immunotherapy targeting TIGIT relies on the cytotoxicity of T/NK cells and thus is ineffective in some ‘cold’ TMEs due to the absence of sufficient TILs. Local injection of drugs to induce inflammation in tumor tissue, and cancer vaccines, can be considered to be combined with IC blockade to enhance therapeutic efficacy [115].
